# Interrelation of Diet, Gut Microbiome, and Autoantibody Production

**DOI:** 10.3389/fimmu.2018.00439

**Published:** 2018-03-06

**Authors:** Ioanna Petta, Judith Fraussen, Veerle Somers, Markus Kleinewietfeld

**Affiliations:** ^1^VIB Laboratory of Translational Immunomodulation, Center for Inflammation Research, Hasselt University, Diepenbeek, Belgium; ^2^Biomedical Research Institute, Hasselt University, and School of Life Sciences, Transnationale Universiteit Limburg, Hasselt, Belgium

**Keywords:** B cells, autoantibodies, diet, microbiome, multiple sclerosis, experimental autoimmune encephalomyelitis

## Abstract

B cells possess a predominant role in adaptive immune responses *via* antibody-dependent and -independent functions. The microbiome of the gastrointestinal tract is currently being intensively investigated due to its profound impact on various immune responses, including B cell maturation, activation, and IgA antibody responses. Recent findings have demonstrated the interplay between dietary components, gut microbiome, and autoantibody production. “Western” dietary patterns, such as high fat and high salt diets, can induce alterations in the gut microbiome that in turn affects IgA responses and the production of autoantibodies. This could contribute to multiple pathologies including autoimmune and inflammatory diseases. Here, we summarize current knowledge on the influence of various dietary components on B cell function and (auto)antibody production in relation to the gut microbiota, with a particular focus on the gut–brain axis in the pathogenesis of multiple sclerosis.

## Introduction

B cells are involved in humoral and cell-mediated immunity. They secrete antibodies following differentiation into plasma cells, produce cytokines, and regulate T cell responses *via* antigen presentation and costimulation (1–3). B cells develop in the bone marrow from hematopoietic stem cells to immature B cells that further mature in the periphery into transitional and mature naïve B cells (4). Following activation, short-lived plasma cells are generated that produce low-affinity immunoglobulin (Ig)M antibodies for a few days (4). A fraction of the responding B cells undergoes a germinal center response, which results in the generation of memory B cells and long-lived Ig class-switched plasma cells that produce high-affinity IgG, IgA, or IgE antibodies.

Autoantibodies can originate from autoreactive B cells that escape tolerance mechanisms following molecular mimicry of infectious antigens with autoantigens, bystander activation, novel autoantigen presentation, or recognition of circulating autoantigens. They can clear target cells *via* antibody-dependent cell-mediated cytotoxicity or complement activation (5, 6). In addition, B cells are highly effective antigen-presenting cells, effectively activating antigen-specific CD4^+^ T helper (Th) cells (2, 7). Depending on the cytokine profile, B cells can stimulate pro- and anti-inflammatory immune responses (8–10).

The humoral immune response in the gastrointestinal tract is mediated by IgA^+^ memory B cells and IgA-producing plasma cells in the gut-associated lymphoid tissue (GALT). The protective and nutrient-rich environment of the gastrointestinal tract accommodates an extremely dense and diverse bacterial community (11) that in turn provides metabolic advantages and serves as a natural defense against colonization with pathogens (12, 13). Commensal bacteria act as critical stimuli, playing an important role for the maturation of the GALT and further induce IgA production by B cells (14). Class switching to IgA-producing plasma cells occurs in the Peyer’s patches and lamina propria, following T cell-dependent or -independent mechanisms (15). The secreted IgA (SIgA) into the gut provides a first-line defense against pathogens mainly by blocking toxins and pathogens from adhering to the intestinal epithelium at the earliest steps of the infection process (16).

In this review, we describe the interrelation of dietary components, microbiome and B cell function with a focus on the production of (auto)antibodies. Special emphasis is placed on multiple sclerosis (MS) and its animal model experimental autoimmune encephalomyelitis (EAE).

## Dietary Influences on B Cell Homeostasis and Function

**Figure 1:**
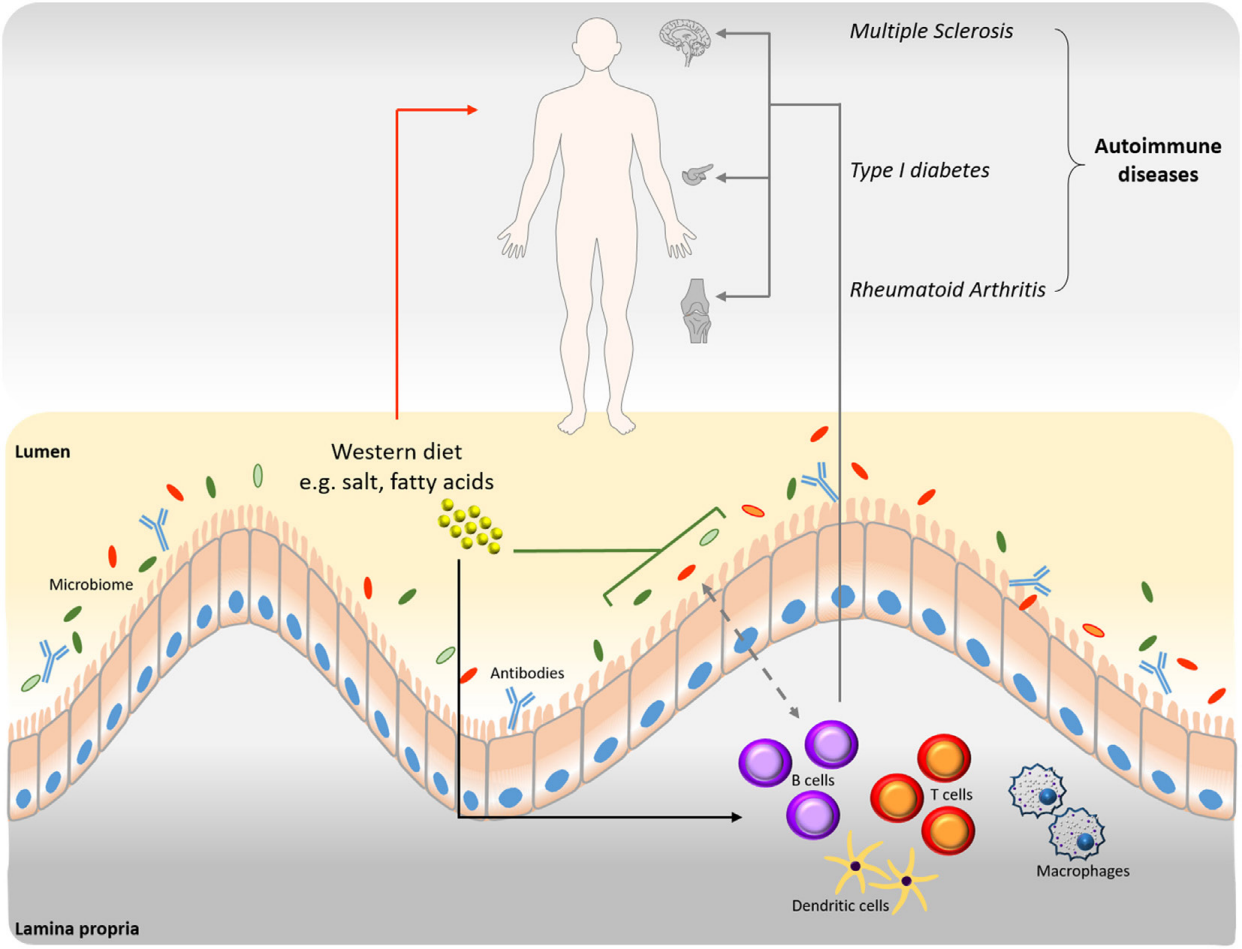
Interrelation among B cells, microbiome, and diet in disease progression. Western type nutritional patterns influence the composition of the intestinal microbiome (green line). Alterations of the gut microbiome induced by nutrient components impact homeostasis and the onset of various diseases (red arrow). Western diet dietary components influence B cell function and production of autoantibodies (black arrow), which are involved in disease progression (gray arrows). The connection between B cells and microbiome is bidirectional (dashed gray arrow). B cell-derived antibodies modulate the intestinal microbiome and *vice versa*.

Modern nutritional patterns, collectively termed “Western-diet,” are characterized by high energy density, animal protein, total and saturated fats, sugars and salt but low levels of plant-derived fibers. This “Western-diet” has a profound influence on the prevalence of autoantibodies, although changes in antibody-independent B cell functions have been reported as well. Additionally, a “Western-diet” may influence the balanced composition of the gut microbiome leading to perturbed immune responses, including effects on B cell production, activity, and maturation (17, 18) (Figure 1).

Effects of a high-fat diet (HFD) on B cell function have mostly been studied in diet-induced obesity models. Here, B cells contribute to pro-inflammatory reactions in the adipose tissue mediating insulin insensitivity and diminished glucose clearance. More specifically, B cells secrete pathogenic IgG antibodies and pro-inflammatory cytokines, which could interfere with macrophage polarization, CD4^+^ T cell function, such as regulatory T cell inhibition or Th17 cell polarization, and CD8^+^ T cell activation (19, 20). Reduced systemic antibody production has been demonstrated following influenza infection and/or *ex vivo* stimulation in a HFD-induced obesity mouse model and in obese individuals (21–23). Underlying mechanisms could involve effects on the responding plasma cells and molecular deregulation. Yet, autoreactive and pro-inflammatory antibodies were increased in obese humans and HFD-fed mice (20, 24, 25), probably through CD40 ligand (CD40L) signaling. CD40L has been shown to induce inflammatory cytokine production in adipose cells *in vitro* and *in vivo* (26, 27). The increased natural autoreactive IgM antibodies under HFD formed an immune complex with apoptosis inhibitor of macrophage, which promoted IgG autoantibody production (28). Increased B cell frequencies and IgG levels were found in mouse obese white adipose tissue and obese humans, who additionally demonstrated a positive correlation between IgM levels and body mass index (21). Furthermore, obese humans displayed reduced IL-10^+^ regulatory B cell levels in subcutaneous adipose tissue, which could contribute to the occurrence of autoantibodies (29). Mouse models further indicated diverse roles for different B cell subtypes in obesity-associated pro-inflammatory responses (20, 29–31). Thus, B cells might play a crucial role in secondary inflammation following obesity and constitute a potential therapeutic target in diet-induced obesity.

High-fat diet also induces changes in the gut microbiota that are related to the development of obesity and diabetes. Obesity is associated with a decreased intestinal abundance of *Bacteroidetes* and an increased proportion of *Firmicutes*, both in mice and humans (32–34). It has been shown that germ-free (GF) mice may be protected against diet-induced obesity and the mechanism possibly involves the fasting-induced adipose factor (Fiaf) in the intestinal epithelium and the AMP-activated protein kinase in skeletal muscle and liver (35). In addition, colonization of GF mice with microbiota harvested from conventionally raised animals resulted in a 60% increase in body fat content and insulin resistance (36). However, more research is necessary to unravel the link between HFD-mediated alterations of gut microbiota and B cell function or autoantibody production.

Another factor associated with “Western-diet” is the high salt content in processed foods and so-called “fast foods.” High salt diets have been shown to exert profound effects on animal models of autoimmunity by affecting Th cell populations and macrophages promoting a pro-inflammatory environment (37, 38). However, if high salt also affects B cells is less well understood. Hybridoma cells under hyperosmotic stress exert suppressed cell growth but enhanced specific antibody production (39–41). Increased differentiation of Th17 and follicular helper T (Tfh) cells was demonstrated following a high salt diet in EAE and a lupus mouse model (42, 43). Tfh cells are involved in the selection of high-affinity B cells during the germinal center response. The mechanism involved in the high salt-mediated Th17 activation is dependent on nuclear factor of activated T cells 5 (NFAT5), p38/MAPK, and the serum/glucocorticoid-regulated kinase 1 (SGK1). SGK1 expression is induced upon salt treatment and its activation depends on p38/MAPK. Silencing of SGK1 reverts the effect of salt on IL-17 levels. To exclude the possibility that high osmolarity mediates the enhanced Th17 pro-inflammatory profile, mannitol and MgCl_2_ were tested along and proved to have only a slight effect (42). Furthermore, high salt conditions result in cellular osmotic stress that is regulated *via* the guanine nucleotide exchange factor Brx-induced expression of NFAT5 (44). Interestingly, Brx was shown to be necessary for B cell differentiation in high salt conditions *via* NFAT5-mediated production of B cell activating factor (BAFF) that regulates splenic B cell differentiation and Ig production. A recent study described the correlation between salt intake and gut microbiome changes in EAE. More specifically, salt intake decreased the population of *Lactobacillus murinus*, while supplementation of *L. murinus* reduced the salt-induced EAE clinical scores and Th17 cell frequencies (45).

By contrast, dietary supplementation with *n* − 3 polyunsaturated fatty acids (PUFAs) derived from fish oils could impact B cell function and suppress pro-inflammatory responses (46). Results from mouse models for obesity, colitis, peritonitis, and systemic lupus erythematosus indicated that dietary administration of fish oil containing n-3 PUFAs elevated splenic B cell numbers, increased B cell cytokine and IgM production while reducing autoantibodies (47–51). Monthly consumption of fish oil by postpartum women led to lower levels of anti-thyroid autoantibodies (52). In individuals at risk for rheumatoid arthritis, the use of n-3 PUFA food supplements and n-3 PUFA levels in red blood cell membranes were inversely associated with anti-cyclic citrullinated peptide and rheumatoid factor positivity (53, 54). Specialized pro-resolving lipid mediators (SPMs) that are endogenously synthesized from n-3 and n-6 PUFAs play a role in suppressing adipose tissue inflammation. In obese humans, selected SPMs were declined in adipose tissue (55). 17-hydroxydosahexaenoic acid (17-HDHA), DHA and resolving D1 stimulated increased Ig production in humans or mice with diet-induced obesity (21, 56). Furthermore, DHA and eicosapentaenoic acid (EPA) induced differential effects on B cell cytokine production and on distinct B cell subtypes that correlated with increased natural serum IgM and cecal IgA in murine obesity (57). Opposed to this, lipoxin A4 decreased (antigen-specific) IgM and IgG production and inhibited memory B cell function in an ovalbumin immunized mouse model (58). Thus, *n* − 3 PUFAs and their derived SPMs can have profound effects on B cell function. More research is needed to clarify the differential effects associated with different types of PUFAs and to mechanistically link the effects to inflammation in obesity. Of note, in MS, EPA and DHA had no beneficial effects on disease activity (OFAMS study) (59).

Furthermore, a diet rich in short-chain fatty acids (SCFAs) could positively impact gut microbiota and inflammatory processes (37). The microbiome converts non-digestible carbohydrates (dietary fibers) to SCFAs, including acetate, butyrate, and propionate, which reduce the risk of inflammatory diseases, type 2 diabetes, obesity, heart disease, and other conditions (60). Non-obese diabetic mice on a diet rich in acetate were characterized by decreased IL-12-producing marginal zone B cells, a B cell subtype linked to the disruption of immune tolerance, in the spleen and the Payer’s patches that additionally showed decreased expression of major histocompatibility complex I and CD86 (61). At the transcriptional level, changes were detected in genes associated with B cell costimulation, antigen presentation, proliferation, and differentiation. Thus, SCFAs and in particular acetate could affect the ability of B cells to expand autoreactive T cells *in vivo* and the development of type 1 diabetes. Butyrate was also suggested to protect against the development of anti-islet cell autoantibodies involved in type 1 diabetes (62). Early introduction of a non-milk diet in infants increased the risk for autoantibody production by reduced butyrate production and was associated with high *Bacteroides* levels. A milk-based diet resulted in a competitive advantage of acetogens compared to sulfate reducing bacteria, thereby leading to increased butyrate production *via* co-fermentation of acetate.

Dietary components such as gluten (63, 64), selenium (65), and iodine [reviewed in Ref. (66)] have been shown to increase autoantibody production. Additionally, impaired protein intake alters IgA responses, attenuating the protective efficacy of vaccination against cholera and *Salmonella enterica* serovar Typhimurium in mice (67). On the contrary, a cocoa-rich diet decreases autoantibody production and confers beneficial immune function (68–72).

## Cross-Talk Between Microbiome and B Cells

Studies in various animal models of impaired microbial control [including GF, antibiotic-treated mice, mice with restricted flora and activation-induced cytidine deaminase knockout (AID^−/−^) mice], but also in humans, have demonstrated that gastrointestinal bacteria participate in B cell differentiation, maturation, and activation (73–76). A proof-of-principle study in Pakistani infants living in impoverished areas showed an accelerated maturation of the salivary IgA system compared with healthy Swedish infants (77). In contrast, in Swedish infants, the gut microbiota took longer to establish and was characterized by a lower diversity (78–80). B cell maturation in Swedish infants was shaped by the intestinal bacterial colonization pattern, mainly by *Escherichia coli* and *Bifidobacteria* (74).

On the other hand, intestinal IgA can influence the gut microbiota composition. Natural and specific IgA antibodies in breast milk were capable of binding commensal bacteria and might be involved in establishing the newborn’s microbiome (81). High-affinity IgA, generated *via* T cell-dependent mechanisms, was essential in mice for the protection from invasive commensal species, such as segmented filamentous bacteria (SFB), and from true pathogens, such as *Salmonella typhimurium* and *Enterobacter cloacae* (82). Of note, SFB increased IgA^+^ B cells *in vivo* (83, 84). Moreover, SIgA promoted the establishment of host–microbial relationships by modulating bacterial epitopes and modifying bacterial metabolism, as demonstrated by the downregulation of bacterial genes involved in the metabolism of oxidative products, i.e., *Bacteroides thetaiotaomicron* (85). An alternative mechanism proposed a mouse monoclonal IgA which was reactive against multiple commensal but not beneficial bacteria by specific recognition of an epitope in serine hydroxymethyltransferase, a bacterial metabolic enzyme (86). Oral administration of this IgA antibody *in vivo* effectively prevented the development of colitis in several mouse models (86, 87).

Probiotics, live microbial food ingredients, have been demonstrated to affect B cell function by stimulating systemic and mucosal IgA production in humans (88, 89). More specifically, probiotic strains such as *Bifidobacterium lactis* and *Saccharomyces boulardii* enhanced IgA production through alteration of the gut mucosa cytokine milieu in preterm infants and mice, respectively (90, 91). Probiotic bacteria can induce TGF-β, IL-10, and IL-6 expression by epithelial cells, which potentiate IgA production through B cell maturation and class switching to IgA (92, 93). Finally, probiotics augment the expression of polymeric Ig receptors on the basolateral surface of intestinal epithelial cells enhancing IgA transcytosis into the gut lumen (94). Not only supplementation of preterm infants with *B. lactis* but also administration of *Lactobacillus casei* in mice resulted in increased IgA-producing cells (95, 96). Pretreatment of mice with the *Bifidobacterium* species *B. bifidum and B. infantis* increased gut mucosal pathogen-specific IgA antibody titers and reduced illness after challenge with rotavirus (97, 98). Similar results were described in infant rabbits and gnotobiotic pigs, pinpointing the effects of several commensals on IgA production (99, 100). Dietary components, shown to directly affect microbiome composition with subsequent influence on human’s immunity and health, include proteins, fats, carbohydrates, and polyphenols. Data from clinical studies prove that plant-derived proteins, non-digestible carbohydrates (prebiotics), and restricted fat consumption and polyphenols increase the intestinal numbers of beneficial bacteria such as *Bifodobacterium* and *Lactobacillus*. Interestingly, contrary to animal-derived proteins that can lead to inflammatory bowel disease and cardiovascular diseases, plant-derived proteins increase SCFAs and regulatory T cells, counteracting inflammatory responses (101).

Therefore, a “Western-diet” lacking components of high nutrient value such as probiotics may negatively modulate immune responses, thereby leading to decreased immune tolerance as well as disease and infection progression.

## Interplay of B Cells and Microbiome in MS

B cells are important players in MS pathogenesis *via* antibody-dependent and -independent mechanisms (1). Bidirectional trafficking of B cells has been demonstrated between the periphery and CNS, where they could locally produce (auto)antibodies (102). IgA antibodies, that mediate humoral immunity in the gastrointestinal tract, have been described to play a role in MS as well. Increased serum IgA antibodies directed against myelin basic protein (MBP), myelin oligodendrocyte glycoprotein (MOG), plant and human aquaporins, and S100B have been described in MS (103, 104). Anti-MBP IgA antibodies were able to catalyze MBP hydrolysis, which could contribute to demyelination (105). Intrathecal IgG, IgA, and IgM synthesis correlated with the presence of anti-MBP or anti-proteolipid protein-secreting cells (106). Interestingly, IgA antibodies were found to be associated with a progressive disease course (103). More specifically, cerebrospinal fluid (CSF) IgA synthesis was correlated with the yearly disease progression rate in primary progressive MS (107). In addition, IgA antibodies directed against gliadin, gluten, and casein were increased in MS patients (108). In the CNS, IgA was reported as a major component of immune responses in MS with IgA^+^ plasma cells showing signs of clonal expansion, intraclonal diversification, and anti-axonal reactivity (109–111). An important correlation was found between CSF levels of chemokine C-X-C motif ligand (CXCL)13 and the extent of intrathecal IgA synthesis (112).

In addition, mounting evidence highlights the implication of the gut environment in MS onset and progression. Recently, microbiome analysis indicated altered levels of several commensals in MS patients (113–115). Possible mechanisms employed by microbiota to induce MS could potentially include low-grade microbial translocation such as peptidoglycan, a bacterial cell wall component, from the gut to the CNS (115, 116). Additionally, gut microbiota can lead to disruption of the blood–brain barrier (117), microglia activation (118), limited astrocyte pathogenicity (119), and expression of myelinating genes (120). Interestingly, microbiota transplantation of MS patients to GF mice resulted in more severe EAE symptoms and reduced IL-10^+^ regulatory T cells compared to mice transplanted with selected healthy human microbiomes (113, 121). Commensal microbiota is necessary for disease development in spontaneous and actively induced EAE models (122, 123). MOG-immunized GF mice showed reduced anti-MOG antibodies that could be increased by colonization with microbiota from MS-affected twins. Furthermore, GF housing conditions resulted in impaired B cell recruitment to brain-draining lymph nodes and reduced MOG-specific IgG2a antibodies in spontaneously developing EAE (121). In line with this, an antibiotic mixture orally administered before EAE induction impaired EAE development due to increased regulatory T cells in the mesenteric and cervical lymph nodes and increased IL-10-producing CD5^+^ B cells in cervical lymph nodes (124). The induced B cells were able to reduce EAE severity when adoptively transferred into naïve recipient mice by causing a shift from a Th1/Th17 toward a Th2 cytokine profile (125). Thus, antibiotic treatment stimulated both regulatory T and B cells, which both contributed to the protection against EAE.

In addition, dietary interventions have been tested in EAE models. A prophylactic diet of 66% caloric restriction protected Lewis rats from developing EAE as evident by reduced splenic CD8^+^ T cells and B cells, lymphoid and thymic CD4^+^ T cells and B cells, and IFN-γ production (126). Other dietary interventions that demonstrated efficacy in reducing EAE symptoms are SCFAs, low fat diets, and zinc aspartate (127). However, currently no information is available on the effects of these dietary components on B cell function or autoantibody production. Moreover, some vitamins, i.e., A, E, and D are important immune regulators and have been shown to limit EAE progression (116). Of note, vitamin D, which is mainly produced by sun exposure but is also contained in food such as salmon, beef meet, and egg yolks, has been shown to decrease EAE manifestations *in vivo*. Administration of 1,25-dihydroxyvitamin D_3_ to mice, the active form of vitamin D, prevented EAE development and significantly reduced serum anti-MBP antibody production (128). In MS patients, different dietary interventions have been studied although mostly unsuccessful or causality could not be demonstrated (116). However, preliminary data from a recent study indicated that a fasting mimicking diet or chronic ketogenic diet could be safe, feasible, and potentially effective in MS treatment (129).

## Conclusion

Increasing evidence is being gathered for the interplay between diet, microbiome, and autoantibody production. Deregulation of this system could contribute to different pathologies, including MS. A “Western-diet” consisting among others of high fat and high salt content has been associated with increased autoantibody production, obesity, inflammatory disorders, and autoimmune diseases. Gut bacteria have been shown to modulate B cell differentiation, maturation, and activation with a profound influence on IgA responses (Figure 1). Dietary interventions and the use of probiotics could restore immune deregulation that is seen in case of diet-induced microbiome alterations. They thus may represent valuable tools for improving the treatment of inflammatory and autoimmune disorders. However, more research is needed to clarify the mechanisms underlying the effects of dietary components on autoantibody production and its relation to disease development in order to obtain a more efficient and preventive treatment line. In MS patients, IgA antibodies against several autoantigens have been described. Additionally, a disturbed microbiome has been observed in MS patients and animal studies have supported a possible link between the microbiome and the disease. However, the exact role of diet and the microbiome in B cell-mediated pathology in MS, along with the respective mechanisms, remain to be determined.

## Author Contributions

IP, JF, VS, and MK wrote the manuscript. All authors approved the work for publication.

## Conflict of Interest Statement

The authors declare the absence of any commercial or financial relationships that could be construed as a potential conflict of interest.
